# Reporting bias in clinical trials: Progress toward transparency and next steps

**DOI:** 10.1371/journal.pmed.1003894

**Published:** 2022-01-19

**Authors:** Mayookha Mitra-Majumdar, Aaron S. Kesselheim

**Affiliations:** Program On Regulation, Therapeutics, And Law (PORTAL), Division of Pharmacoepidemiology and Pharmacoeconomics, Department of Medicine, Brigham and Women’s Hospital and Harvard Medical School, Boston, Massachusetts, United States of America

## Abstract

Mayookha Mitra-Majumdar and Aaron Kesselheim reflect on steps taken to combat reporting bias in clinical trials over the last two decades.

Medical guidelines and decision-making are informed by an understanding of publicly reported clinical trial results, with the assumption that the evidence base is complete and reported in a way faithful to actual trial outcomes [1]. However, studies suggest that reporting bias—in which the outcomes of a trial affect whether and how its results are published—is not uncommon [2]. Reporting bias can skew the perceived risk–benefit ratio of treatments, mislead medical professionals and policymakers, and ultimately result in suboptimal medical decisions. A seminal 2008 study from Turner and colleagues investigated reporting bias in the publication practices of clinical trials supporting 12 antidepressants approved by the United States Food and Drug Administration (FDA) between 1987 and 2004 [3]. The results showed that trials judged to show a positive effect by the FDA were 12 times as likely to be published in a way consistent with the FDA analyses as were studies with nonpositive results according to the FDA. As a result, published studies suggested that drugs had an effect size nearly a third greater than the effect size derived from the FDA data. In an accompanying study in *PLOS Medicine*, Turner and colleagues revisit the antidepressant space to study whether the prevalence of 2 types of reporting bias—study publication bias and outcome reporting bias—among the clinical trials of 4 recently approved antidepressants reflects progress toward greater reporting transparency [4].

Study publication bias occurs when trials showing negative or no effect are not published; outcome reporting bias occurs when authors fail to report unfavorable data, include only a subset of data analyzed, or change or omit the outcome of interest in the interest of statistical significance [5]. Factors that drive reporting bias include journals’ preferences (real or perceived) for studies showing more “exciting” results (i.e., positive outcomes, nonzero effect sizes, and statistically significant findings) and financial incentives for brand name pharmaceutical manufacturers to publish favorable data on their drugs to increase the likelihood of uptake in clinical practice. The issue is widespread: A meta-analysis of more than 4,600 papers published between 1990 and 2007 found that the odds of reporting a positive result (i.e., outcomes supporting the study hypothesis) increased by 6% annually [6]. Since then, other reviews have found similar trends in other fields, such as oncology [7].

In the past 20 years, however, awareness of the prevalence and consequences of reporting bias has spurred efforts toward greater transparency. The International Committee of Medical Journal Editors announced that, beginning in 2005, prospective trial registration would be a prerequisite for publication in its member journals [8]. In the same year, the World Health Organization launched its International Clinical Trials Registry Platform to enhance public access to information [9]. In 2007, the Food and Drug Administration Amendments Act (FDAAA) section 801 mandated clinical trial registration and results reporting on ClinicalTrials.gov, a registry established by the National Institutes of Health (NIH) in 2000. Trial sponsors must also post summary results of certain clinical trials within 12 months of completion and are subject to up to a US$10,000 fine each subsequent day until they comply.

In their new paper, Turner and colleagues examine reporting bias among the 30 randomized, double-blind, and placebo-controlled Phase II and III trials that supported 4 recent antidepressant approvals—desvenlafaxine (approved February 2008), vilazodone (January 2011), levomilnacipran (July 2013), and vortioxetine (September 2013). Of the 30 trials, the FDA deemed 15 to yield statistically significant findings on the prespecified primary outcome. All of these studies were published in accordance with the FDA’s report. Of the 15 nonpositive trials, 7 (47%) were transparently published as nonsignificant, greater than that observed for the cohort examined in their 2008 paper (11%). The remainder were either not published or not transparently published. Controlling for trial outcome, trials in the newer cohort were 6.6 times more likely to be transparently reported than those in the older cohort (odds ratio 6.6; 95% CI 1.6 to 26.4, *p* = 0.008), almost entirely due to more transparent reporting of nonpositive trials. While the authors acknowledge certain limitations in their methodology—for example, safety data were omitted from their transparency analysis, and the last drug approval date was 2013, precluding any conclusions on the impact of more recent transparency efforts—these results are cautiously optimistic.

Other studies also indicate progress in reporting transparency. A 2017 study of cardiovascular and diabetes drugs approved between 2005 and 2014 showed that of 183 supporting Phase II and III clinical trials identified in drug approval packages, 83% (151/183) were registered on ClinicalTrials.gov, 54% (99/183) had posted results, and 92% (169/183) were published in a peer-reviewed journal [10]. However, there is still work to be done. The same study found that 97% of post-FDAAA published trials (74/76) were published transparently compared to 84% (78/93) of pre-FDAAA published trials [10]. Thus, FDAAA may have contributed to a move toward greater transparency, but there is still a need to address selective registration, publication, and outcome reporting on ClinicalTrials.gov for pre-FDAAA trials. A final rule amending FDAAA section 801 took effect in 2017, expanding the number of data elements sponsors are required to submit and setting time limits for corrections. The NIH has since reported that clinical trial registration and results submissions have grown since the final rule took effect, with continued increases expected with ongoing outreach and education. However, the NIH and the FDA have rarely taken action against noncompliant clinical trial sponsors—there are over US$5 billion in accrued FDAAA fines, suggesting an opportunity for more rigorous enforcement [11].

The publishing industry too must strive to improve. Journals, even those that support preregistration of trials before publication, continue to publish unregistered or late registered trials [12]. Contemporary efforts such as the Centre for Evidence Based Medicine Outcome Monitoring Project (COMPare) have also investigated discrepancies between published studies in top 5 general medical journals (*New England Journal of Medicine*, *The Lancet*, *JAMA*, *BMJ*, and *Annals of Internal Medicine*) and their trial protocols [2]. COMPare found that only 13% (9/67) of clinical trials published between October 2015 and January 2016 had the same primary and secondary outcomes across protocols, registries, and articles. When COMPare reached out to the journals about this issue, only *BMJ* and *The Lancet* agreed to publish corrections acknowledging the discrepancies.

An emerging peer review model, Registered Reports, may be one way to disincentivize behaviors that contribute to reporting bias [13]. Authors first submit an initial manuscript based on pilot studies. After editorial and peer review and requested author revisions, manuscripts are offered in principle acceptance (IPA) based on the rigor of their study design, rather than study results. Efforts such as COMPare and Registered Reports signal continued action in the research community to mitigate reporting bias. We should take stock of the progress that has been made and continue to build on those efforts to ensure that publicly available trial information is maximally useful to the medical community and policymakers. Better enforcement of registration requirements, in conjunction with independent efforts from the research community, can continue to promote transparency of data to allow medical professionals to make more informed prescribing decisions in the best interest of their patients and public health.

## Abbreviations

COMPare: Centre for Evidence Based Medicine Outcome Monitoring Project
FDA: Food and Drug Administration
FDAAA: Food and Drug Administration Amendments Act
IPA: in principle acceptance
NIH: National Institutes of Health
